# Supplementary material for: Relationship between Dietary n-6 Fatty Acid Intake and Hypertension: Effect of Glycated Hemoglobin Levels
Source: Nutrients. 2018 Nov 24;10(12):1825. doi: 10.3390/nu10121825 (PMC6316654; doi:10.3390/nu10121825)
Supplement: Supplementary file 1 [file nutrients-10-01825-s001.pdf]

Supplementary Table S1. Interaction between BP and gender groups by n-6 fatty acid intake.

n-6 fatty acid		Hypertension	Normal BP	
	HbA1c level	Average (SD)	Average (SD)	P value for interaction
n-6 fatty acid (g/1000kcal)	Male	4.77 (1.31)	5.18 (1.41)	0.312
	Female	5.56 (1.46)	5.75 (1.25)	
LA (g/1000kcal)	Male	4.62 (1.28)	5.02 (1.39)	0.309
	Female	5.40 (1.43)	5.58 (1.23)	
GLA (mg/1000kcal)	Male	3.78 (2.44)	3.91 (2.45)	0.700
	Female	4.16 (2.36)	4.46 (3.24)	
EA (mg/1000kcal)	Male	22.4 (8.32)	24.4 (8.23)	0.452
	Female	25.4 (8.69)	26.3 (8.38)	
DGLA (mg/1000kcal)	Male	14.5 (5.18)	15.3 (4.55)	0.966
	Female	16.6 (5.05)	17.4 (4.91)	
AA (mg/1000kcal)	Male	86.3 (32.2)	91.0 (30.2)	0.649
	Female	95.7 (30.5)	98.1 (28.4)	
DPA (mg/1000kcal)	Male	5.12 (3.16)	4.97 (2.98)	0.652
	Female	5.63 (3.24)	5.24 (3.53)	

P values from two-way analysis of variance means interaction between gender groups and BP groups. Hypertensive subjects were defined as participants with a greater SBP than 140 mmHg, a DBP than 90 mmHg, or the use of antihypertensive medication. 299 participants were male; 103 male participants had a normal BP, and 196 had hypertension. There were 334 female participants; 180 had a normal BP, and 154 had hypertension. Abbreviations: BP, blood pressure; HbA1c, hemoglobin A1c; LA, linoleic acid; GLA. Gamma-linolenic acid; EA, eicosadienoic acid; DGLA, dihomo-gamma-linolenic acid; AA, arachidonic acid; and DPA, docosapentaenoic acid.
